# Evaluating the Effects of Camellia Sinensis (Green Tea) and Teucrium Polium Extracts on Salivary *Streptococcus Mutans* Levels in Children

**DOI:** 10.30476/DENTJODS.2021.92379.1640

**Published:** 2023-03

**Authors:** Fatemehsadat Sajadi, Mahboobeh Shokrizadeh, Maryam Sharifi, Reyhaneh Aftabi

**Affiliations:** 1 Oral and Dental Diseases Research Center, Kerman Social Determinants on Oral Health Research Center, Dept. of Pediatric Dentistry, School of Dentistry, Kerman University of Medical Sciences, Kerman, Iran; 2 Pediatric Dentist, Kerman University of Medical Sciences, Kerman, Iran; 3 Kerman Social Determinants on Oral Health Research Center, Oral and Dental Diseases Research Center, Department of Pediatric Dentistry, School of Dentistry, Kerman University of Medical Sciences, Kerman, Iran; 4 Postgraduate Student, Dept. of Pediatric Dentistry, School of Dentistry, Kerman University of Medical Sciences, Kerman, Iran

**Keywords:** Dental Caries, Camellia sinensis, Real-Time Polymerase Chain Reaction, Streptococcus mutans, Teucrium

## Abstract

**Statement of the Problem:**

*Camelia Sinenis* or green tea (GT) and *Teucrium polium* (TP) are known to have a great antimicrobial potential on salivary *Streptococcus mutans* (*S. mutans*). Their efficacy should be examined compared to the gold standard antimicrobial agents.

**Purpose:**

To evaluate the effects of *Camelia Sinenis* or green tea (GT) and *Teucrium polium* (TP) extracts in comparison with chlorhexidine gluconate (CHG) on salivary *S. mutans* levels.

**Materials and Method:**

This double-blinded randomized clinical trial study was conducted on 90 preschool children aged 4 to 6 years and assigned randomly (simple randomization) to three groups as GT, TP, and CHG. Unstimulated saliva samples were then collected in three times as before application of agents, after half an hour, and after one week. To determine *S. mutans* levels, quantitative polymerase chain reaction (qPCR) technique was additionally utilized. Statistical analysis was also fulfilled using Shapiro-Wilk test, Friedman test, Chi-square test, paired sample t-test, repeated measures analysis of variance (ANOVA), and Mann-Whitney U test at a significance level of 0.05.

**Results:**

The results of this study established a significant difference between mean salivary *S. mutans* levels after administration of the three compounds. Although the mean of *S. mutans* levels reduced significantly following the application of CHG and TP after half an hour, the mean salivary *S. mutans* levels in the group receiving GT declined in a significant manner only one week later (*p*< 0.05).

**Conclusion:**

The results of this study indicated that GT and TP extracts had considerable effects on salivary *S. mutans* levels compared with CHG.

## Introduction

Dental caries or tooth decay is a common and chronic infectious disease in children and different types of bacteria have been introduced as caries-producing microorganisms [ 1 ]. In this respect, *Streptococcus mutans* (*S. mutans*) is known as the most important microorganism observed at the onset of decay; however, *Lactobacillus acidophilus* (*L. acidophilus*) is the most frequent one affecting progression of dental caries [ 2 ]. 

It is noteworthy that early childhood caries (ECC) is a significant public health problem in both developed and developing countries. ECC can commence early in childhood, progress rapidly, affect children’s quality of life, and influence social aspects [ 3 - 5 ]. 

Chlorhexidine gluconate (CHG) is considered as the gold standard among antibacterial agents [ 6 ]. It is also a bisguanide known for its significant bacteriostatic and bactericidal features. This agent has been proven effective in prohibiting plaque formation and counteracting a wide spectrum of bacteria and viruses [ 6 ]. Although CHG is being broadly used, it causes mitochondrial dysfunctions (which leads to decreasing cell viability and cytotoxicity), extrinsic staining, mild mucosal irritation, alteration in taste perception, and rarely increased calculus formation [ 7 - 8 ]. 

Over recent years, there is an increasing interest in using natural extracts in medicinal antimicrobial materials to replace synthetic ones. Plants can produce some compounds to protect themselves against pathogens, serve as antimicrobial effects [ 9 ], and have numerous benefits such as availability, less toxicity, and low cost [ 10 ].

Tea, which appears as white, green (GT), oolong, and black, is provided by mature unfermented leaves [ 11 ]. Several health benefits have been accordingly highlighted for the use of GT such as anti-inflammatory effects, anticarcinogenic impacts by inducing apoptosis in cancer cells, cardio-protective, cholesterol lowering, anti-hypertensive, anti-diabetic, and antimicrobial effects in oral health, as well as synergic impacts with other antimicrobials [ 12 , 13 ]. GT has also a direct effect on *S. mutans* and it seems to prevent its attachment to both teeth and gum [ 1 ]. Moreover, this type of tea can be a natural source of fluoride. Moreover, it seems to have antimicrobial effects on some fungi like *Candida albicans* and some common viruses such as *herpes simplex virus* (HSV), *human immunodeficiency virus* (HIV), and *influenza* [ 14 ]. In this line, Anita *et al*. [ 15 ], Salama *et al* [ 16 ], and Arab *et al* [ 17 ] indicated that GT could have antibacterial effects on predominant cariogenic bacteria including *S. mutans* and *L. acidophilus*. According to Ardakani *et al*. [ 18 ], although the mouth rinse made with chamomile extract had revealed an *in vitro* antibacterial activity inferior to 0.2% CHG, the pure extract had significant bactericidal effects. 

Teucrium polium (TP), which is colloquially known as *Kalpooreh* in Iran, has been already introduced as an important medicinal plant with extensive effects [ 9 ]. Studies have additionally demonstrated anti-oxidative, antiplatelet, disinfectant, bactericidal, antifungal, antipyretic effects of TP; although few investigations have reported the antibacterial effects of TP on *S. mutans* and *L. acidophilus* [ 2 , 19 - 21 ]. Belmekki *et al*. [ 22 ] had also concluded that TP essential oil had antimicrobial effects, which could be applied as a natural antimicrobial agent. Otman *et al*. [ 23 ] had proved that TP contained bioactive compounds. Consequently, this plant had been introduced to have antifungal and antibacterial properties in its ethanol extract and essential oil. 

There are several methods for characterizing salivary *S. mutans* levels [ 24 ]. Traditionally, culture-based methods for detecting and measuring *S. mutans* levels have been among the most common ones. The rate of *S. mutans* colonization can be also measured through colony-forming unit per millimeter (CFU/ml) [ 24 ]. However, this method is difficult to implement with regard to its result interpretation and it has low sensitivity in terms of bacterial colony identification [ 24 ]. Therefore, polymerase chain reaction (PCR) has been developed as an advanced molecular assay method to identify oral bacteria; it is a culture-independent procedure and is more sensitive in characterizing bacterial deoxyribonucleic acid (DNA) than mere culture [ 24 ]. In order to explore more reliable application of this method, some changes including quantitative fluorescence PCR (qPCR) are added. The most advantages of this technique include reliable sensitivity, a better accuracy in enumeration, and good versatility of obtaining results [ 27 - 28 ].

The main approach of this study is to assess the antibacterial activity of GT as well as TP extracts in comparison with CHG on salivary *S. mutans* levels.

## Materials and Method

### Ethical Considerations

The ethical code of this double-blinded randomized clinical trial is IR.KMU.REC.1395.681 along with Iranian Registry of Clinical Trials (IRCT) registration number: IRCT20171020036896N5. This investigation is carried out according to the regulations of the World Medical Association Declaration of *Helsinki*.

### Participants

In the current investigation, ninety (4-6 year-old participants) were selected by means of exclusion and inclusion criteria. The reason for selecting this age group was that the children in this age group could learn the procedure of spilling out the saliva. This research was performed at the Department of Pediatric Dentistry in Kerman University of Medical Sciences, Iran. 

Inclusion criteria were good oral hygiene (brushing at least daily), good physical health status, no systemic diseases, no previous antibiotic Xylitol-containing lozenges and chicle gum usage, as well as no history of receiving fluoride mouthwash or varnish for at least one month before sampling. As well, exclusion criteria were defined as presence of rampant caries, soft tissue lesions, cold sores, recurrent aphthous stomatitis (RAS) and localized/ generalized active periodontitis as well as children with a history of usage of any medication or antimicrobial mouthwash like CHG and children who had prosthetic-orthodontic appliances [ 27 - 28 ]. 

### Plant Extract Preparation

The fresh leaves and flowers of GT and TP were harvested from the tea gardens in Lahijan, and cultivated hills around Kerman cities, Iran. For preparing 5% methanolic GT and methanolic TP gel, the leaves were sent to the herbarium laboratory of Pharmacognosy department of Faculty of Pharmacy, Kerman University of Medical Sciences, Kerman, Iran, where the voucher specimens were preserved as VGT-2005 and VTP-1249, respectively.

The dried parts of these medicinal plants were initially grinded to make a fine powder. This was followed by soaking 100 gr of GT and TP powder in 500 ml of methanol (alcohol) for 48 hours, then the solution was filtered and the residue was transferred into plates at room temperature for 4 days. Afterwards, solid powder was mixed the Carbopol-934 base gel. The 5% methanolic gel of GT and TP was prepared by adding 5 g of the dried extract of each of them to 100 g of Carbopol-934 as the gel base [ 26 ]. Ultimately, each gel was kept at 0 and 4°C. All the agents were also prepared in the form of a gel for easy application [ 9 , 21 ].

### Sampling

Sample size was estimated 90 children, according to sample size formula 

$n=\frac{{\left(Z_{1-\frac{\alpha }{2}}+Z_{1-\beta }\right)}^{2}(S_{1}^{2}+S_{2}^{2})}{d^{2}}$

considering type I error 5% and test power 90%. 

### Randomization and Intervention

A double-blinded procedure was employed in this study. All the materials were in form of gels with the same consistency in the sample container, covering the color of the gels. For being blinded to the odor, the researcher in change of sampling was trained how to use a mask during applying each of them and also to change it following sampling for each group. Each group was also assigned with a code. Then, written informed consent was obtained from each parent before screening for gel application. In order to blind the participants, GT and TP gels were prepared in the same consistency, shape, and color. The researcher and patients were blind to study groups. Moreover, the study subjects and the evaluator who performed the qPCR technique were unaware of the mentioned materials and groups.

First, the researcher provided explanations about study aims to parents and then informed consent was obtained from them. The participants were randomized following the simple randomization of Roll of a dice as the method. There were 90 children in this study. The children were classified into three groups. Each group included 30 children. The participants were numbered as 1 and 2, considered as the first group (CHG), numbers 3 and 4 were given to the second group green tea (GT), and numbers 5 and 6 were allotted to the third group (T.P). To meet the allocation concealment criteria, 90 obscured and occasional order envelopes with generated links were used. The researcher also fulfilled oral examinations and was recorded decayed, missing, and filled teeth (dmft) for each child, regarding the AAPD definition [ 29 ].

In the beginning of the test, the operator was trained by a supervisor, 25 patients were examined by the supervisor, and operator until the operator was completely trained and calibrated. None of these patients was participated in the study. To be noted that this study only included children with dmft equal or greater than 4, who had at least 3 carious teeth in their mouth. 

Horizontal brushing techniques as well as selective techniques suitable for this age group were taught to parents and asking them to brush their children’s teeth without toothpaste for 2 weeks and not to allow them using Xylitol-bearing gums or CHG and fluoride mouthwashes (considered as a washout period).

During the experiment, the children were assigned to have their breakfast and to wash their mouth without using toothbrush. The participants were not allowed to eat anything else for an hour before unstimulated saliva sampling. During the saliva sampling, children were asked to sit in a comfortable chair at the coachman position.

 In the first step, 0.5 to 1 cc of unstimulated saliva was sampled from every participant in the coachman position and sent to a laboratory to estimate *S. mutans* levels. In the second step, the children rinsed their mouth, and dried with compressed air. The Isolation was achieved by using a cotton roll for each quadrant, and then 1 cc of each gel was randomly applied on all of teeth at the same coachman position. For 30 min, the child was prohibited to eat and drink. Then, the second unstimulated saliva samples were gained as the same as that in previous manner.

The third step was followed by asking the parents to brush their children’s teeth for one week. The third unstimulated saliva sampling was done after one week.

It should be noted that GT and TP were produced by pharmacist, since there was no commercial production for them. Moreover, CHG gel 2% (Cavitec, Ivoclar Vivadent) was used in this study.

An oral microbiologist determined the *S. mutans* levels using qPCR technique [ 29 ]. Firstly, the frozen sample was dissolved at 37°C, then 300μl of saliva sample was used for extraction of the *S. mutans* DNA (ATCC 35668), using a DNA extraction kit (RIBO-prep), following the manufacturer’s protocol. In this regard, the Center for Collection of Industrial Bacteria and Fungi affiliated to Iranian Research Organization for Science and Technology (IROST) provided the standard strain of *S. mutans* (ATCC 35668). In order to count the bacteria, the Applied Biosystem StepOnePlus real-time PCR was used and serial dilutions of 10 were made from standard strain genomic DNA (at a concentration of 9ng/μl). The *S. mutans* bacterium in saliva samples was identified by using the gtfB gene coding with glucosyltransferase activity.

### Statistical Analysis

Data analysis was completed using the IBM SPSS Statistics software (version 22), was fulfilled by Shapiro-Wilk test, Friedman test, Chi-square test, paired sample t-test, repeated measures analysis of variance (ANOVA) and Mann-Whitney U test at a significance level of 0.05.

## Results

Mean salivary *S. mutans* level for CHG was 42.1±10.58 before administration, 14.01±12.62 half an hour and 34.01±12.47 one week after administration. For TP, before administration: 30.06±2.69, half an hour: 16.37± 2.97 and one week after applying: 20.52±2.53. The last compound, GT showed 18.76±9.35 before administration, 17.58±9.05 half an hour after applying and decreased to 11.31±3.51 after one week (Table 1). 

**Table 1:** Mean and standard deviation (SD) of salivary *S.mutans* levels after use of three compounds by time

Compounds	Before administration		Half an hour after administration		One week after administration	
	Mean	SD	Mean	SD	Mean	SD
CHG	42.10	10.58	14.01	12.62	34.01	12.47
TP	30.06	2.69	16.37	2.97	20.52	2.53
GT	18.76	9.35	17.58	9.05	11.31	3.51

CHG= Chlorhexidine gluconate, TP=Teucrium polium, GT=Green Tea,*p< 0.05

**Figure 1:**
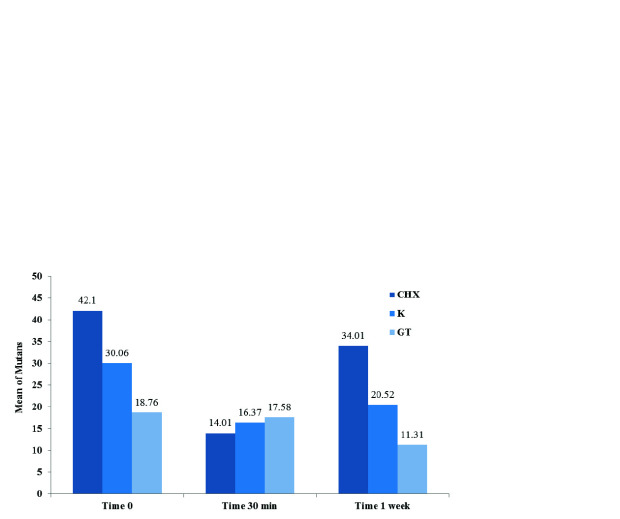
The comparison of *S.mutans* levels at three groups of compounds at three different times

The data display that the mean of salivary *S. mutans* levels in all CHG, TP, and GT compounds had significantly changed over time, so that the mean salivary *S. mutans* levels had declined significantly thirty minutes after following the application of CHG (*p*< 0.001) and TP (*p*< 0.001). Considering the application of GT, *S. mutans* level did not significantly decline half an hour after use (*p*= 0.02), while significantly reduced after one week (Table 2, Figure 1).

**Table 2:** Comparing salivary *S.mutans* levels after use of three compounds at three different times

Compounds	Comparison of administration time (*p* Value)			
	Before administration- Half an hour after administration	Before administration- One week after administration	Half an hour after administration- One week after administration	Mean difference t-test
CHG	<0.001*	0.10	0.008*	<0.001*
TP	<0.001*	<0.001*	<0.001*	<0.001*
GT	0.93	0.04*	0.06	0.02

CHG= Chlorhexidine gluconate, TP=Teucrium polium, GT=Green Tea,

* *p*< 0.05

**Figure 2:**
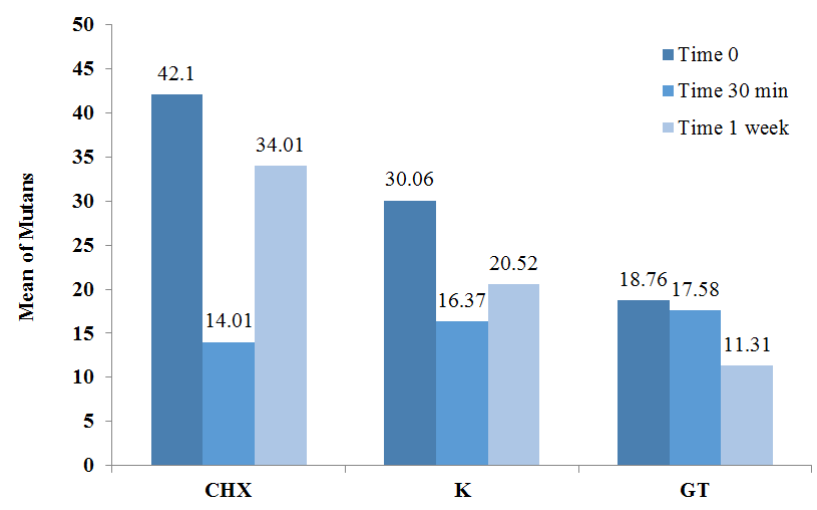
Salivary *S.mutans* levels after use of three compounds at three different times

**Figure 3:**
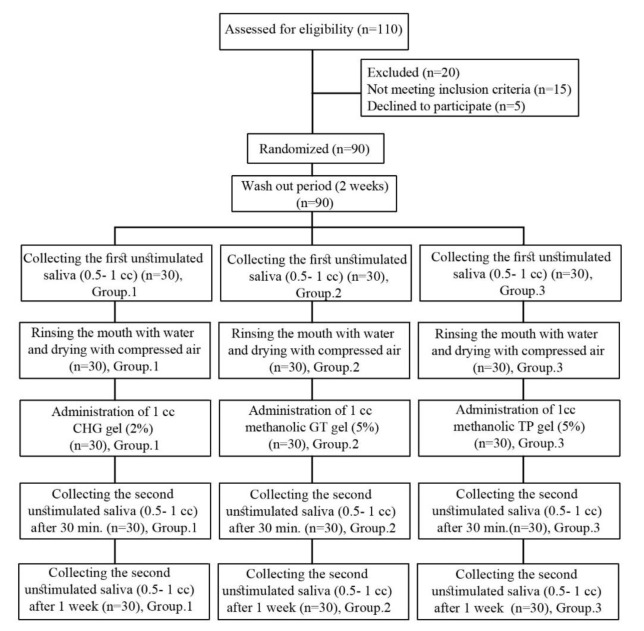
CONSORT flowchart of the participants' progress through the trial

The level of salivary *S. mutans* after using CHG at different times decreased before administration, 30 min after administration significantly (*p*< 0.001). The TP showed a significant decrease in three different times. (*p*< 0.001) Furthermore, GT demonstrated a significant reduction only in period of time, before administration and one week after administration (*p*= 0.04) (Table 2, Figures 2-3).

Kruskal-Wallis test was employed to determine which of the compounds was more effective in lowering the mean salivary *S. mutans* levels at different times. The results revealed no significant difference between the effects of three compounds on bacterial reduction after half an hour (*p*= 0.46). After one week of admin istration, the effects of TP and GT were significantly more effective compared to CHG. Moreover, a significant difference was evident between TP and GT (*p*= 0.002) (Table 3).

**Table 3:** Comparing effects of three compounds on reducing salivary *S.mutans* levels after one week

Time intervals	Comparison of mean difference	*p* Value
One week after administration	CHG-TP	<0.001
	CHG-GT	<0.001
	TP-GT	0.002*

CHG= Chlorhexidine gluconate, TP=Teucrium polium, GT=Green Tea,

* *p*< 0.05.

## Discussion

Dental caries is known as a chronic infection disease and unfortunately, many children are receiving inadequate dental care, so ECC is going to be the single most common childhood disease [ 2 ]. *S. mutans* is the most cariogenic microorganism and has a main role in the beginning and developing of tooth decay [ 1 ]. As well, removal of *S. mutans* can be done through mechanical and chemical methods. In children, lack of dexterity and less motivation for tooth brushing would be the cause for more plaque accumulation as well as an increase in *S. mutans* load. Therefore, chemotherapeutic agents as mouthwashes for plaque control especially at interproximal surfaces are necessary [ 5 - 6 ].

The results of this study suggest that the administration of 2% CHG and 5% methanolic extracts of GT and TP could decrease the mean salivary *S. mutans* levels in thirty minutes and after one week of administration; respectively. Reduction in bacteria count for CHG and TP was also higher than GT in 30 min and well kept their substantivity for one week. However, the reduction of bacterial count for CHG decreased after one week, but the bacterial count was still below the baseline, although it was not significant. Although, after one week, the samples showed a rising trend in bacterial count, it was still significant. The data also indicated that salivary *S. mutans* levels had exhibited some reduction at 30 min after GT administration. Although, in half an hour, this decreasing trend was not considerable, statistically significant reduction was obtained one week later thus; GT needed more time to show its therapeutic effect.

Besides, GT is considered as an effective medicinal plant in preventing dental caries [ 11 ]. This agrees with similar findings suggested by Goyal *et al*. [ 30 ] and Servin *et al*. [ 31 ], thus this type of tea has dual antibacterial and antiplaque effects. Its antibacterial activity refers to flavor compounds and antiplaque effect is the consequence of polyphenol agents. Furthermore, catechins such as epigallocatechin (EGC) and Gallo catechin (GC) are the most common inhibiting factors affecting *S. mutans*. Gallo catechin gallate (GCG), epigallocatechin gallate (EGCG) and EGC block the enzyme guanylyl transferase (GTPase) activity and prohibit attachment of *S. mutans* to dental surface [ 12 , 14 ]. In this respect, Archana *et al*. [ 11 ] found that GT was characterized by antibacterial and antifungal properties. Moreover, Araghizadeh *et al*. [ 14 ] observed that GT showed a significant reduction in aerobic mouth bacterial load, which could prevent plaque formation on teeth. Also, GT mouthwash was regarded as a safe and nontoxic mouthwash particularly for children and pregnant women. They also obtained a significant difference between mouth rinse and water (as a control group) in 2 hours, but in the present study, reduction was observed after 30 min and considerable anti-bacterial effect on *S. mutans* was achieved after one week.

TP is also known as one of the most popular medicinal plants with pharmaceutical uses with anti-inflammatory, antioxidative, antibacterial, and antiviral effects [ 9 ]. Other therapeutic benefits of this plant are its systemic effects including hypolipidemic, hypoglycemic, and antirheumatic effects. More importantly, both methanolic and ethanolic extracts of TP have been reported to influence both gram-negative and gram-positive bacteria [ 20 ]. The given plant also contains a wide variety of metabolites like tannin, sterols, alkaloids, and leucoanthocyanin, having antimicrobial properties [ 21 ]. Moreover, antifungal activities of TP against different species of *Candida albicans* have been identified [ 9 ]. Accordingly, Otman *et al*. [ 23 ] had introduced TP as a plant with bioactive compounds, which might be useful for several activities such as antibacterial and antifungal properties. In the present study, the antibacterial effect of TP was reported more than CHG and GT, which supports similar results given by Khoramian Tusi *et al*. [ 32 ]. Although, the *S. mutans* levels increased after one week, but this increasing rate was still significantly lower than before application of TP. This result can be probably related to long-term effect of TP in compared with CHG. Antibacterial effect of GT after half an hour was not similarly significant compared with that before application, but it significantly reduced the *S. mutans* levels after one week, which might be related to delayed onset effect of GT with possibly long effects. Also, Arab *et al*. [ 17 ] had found that GT products could have positive effects on preventing and curing a variety of oral and periodontal diseases. In addition, Sajadi *et al*. [ 33 ] indicated that the GT had more enduring antibacterial effects compared to the CHG, although, Krishna Priya *et al*. [ 34 ] reported that CHG had a better antibacterial effect compared to the GT.

Our results showed that all of the three materials employed in the current study (GT, TP and CHG) have perfect antibacterial activity in short time, but, the antibacterial effect of GT was not statistically significant in short time. Additionally, TP continued its antimicrobial activity for one week, GT's activity increased significantly after one week , while the antibacterial effect of CHG decreased after one week. In other words, *S.mutans* levels increased after one week, indicating the short-term effect of CHG compared with long-term antibacterial activity of TP and GT. Based on Eascott and Stallard [ 35 ], plaque formation had occurred in 2 hours and by 24 hours and then rod-shaped bacteria had been appeared. In addition, filaments could cover the plaque within 48 hours. According to the present study, CHG could be effective on the first colonizing bacteria, due to its short-term antibacterial effect, whereas the late colonizing bacteria such as filaments could disappear just by using TP, which has a significant long-term antibacterial activity. 

Despite the reliable antimicrobial effects of TP and GT, there were some limitations regarding the children and parents in this study including the unpleasant taste of gels for some children, their unwillingness during saliva collection, and the need for more chair time in the course of saliva collection. Moreover, parents were worried about negative side effects of these herbal extracts and it was difficult to convince them. 

## Conclusion

TP and GT had antibacterial activities against *S. mutans*, thus, both of these agents in the form of topical gels can be considered as effective and cost-effective herbal compounds. Moreover, these productions might be regarded as an additive in the cleaning regime of children.

## Acknowledgments

We sincerely thank Kerman University of Medical Sciences that supported this study. We highly appreciate insightful comments made by Editor-in-Chief and three anonymous reviewers.

## Conflict of Interest

The authors declare that they have no conflict of interest.
